# Experimental Evolution of Anticipatory Regulation in *Escherichia coli*

**DOI:** 10.3389/fmicb.2021.796228

**Published:** 2022-01-11

**Authors:** Anjali Mahilkar, Pavithra Venkataraman, Akshat Mall, Supreet Saini

**Affiliations:** Department of Chemical Engineering, Indian Institute of Technology Bombay, Mumbai, India

**Keywords:** evolution of anticipatory regulation adaptation, anticipatory regulation, *Escherichia coli*, laboratory evolution, rhamnose, paraquat

## Abstract

Environmental cues in an ecological niche are often temporal in nature. For instance, in temperate climates, temperature is higher in daytime compared to during night. In response to these temporal cues, bacteria have been known to exhibit anticipatory regulation, whereby triggering response to a yet to appear cue. Such an anticipatory response in known to enhance Darwinian fitness, and hence, is likely an important feature of regulatory networks in microorganisms. However, the conditions under which an anticipatory response evolves as an adaptive response are not known. In this work, we develop a quantitative model to study response of a population to two temporal environmental cues, and predict variables which are likely important for evolution of anticipatory regulatory response. We follow this with experimental evolution of *Escherichia coli* in alternating environments of rhamnose and paraquat for ∼850 generations. We demonstrate that growth in this cyclical environment leads to evolution of anticipatory regulation. As a result, pre-exposure to rhamnose leads to a greater fitness in paraquat environment. Genome sequencing reveals that this anticipatory regulation is encoded *via* mutations in global regulators. Overall, our study contributes to understanding of how environment shapes the topology of regulatory networks in an organism.

## Introduction

Environmental cues are often cyclical in nature, for instance, temperature in day is higher and night is cooler. Thus, microorganisms have to continuously adapt to this changing environment. Moreover, the changing cues have a strong temporal element associated with them. That is, high temperature precedes the lower temperature of the night. Can the gene expression pattern of a microbial population be tuned to pre-empt the arriving cue? While examples of such anticipatory gene regulation are known from ecological contexts (Tagkopoulos et al., 2008; Mitchell et al., 2009), the dynamics of evolution of anticipatory regulation are not known.

The cellular response to a biological cue is based on specificity of recognition of the cue. Biological systems expend considerable effort toward reducing non-specific substrate binding and reducing crosstalk (Swain and Siggia, 2002; Siryaporn and Goulian, 2008; Skerker et al., 2008; Johnson and Hummer, 2011). However, anticipatory regulation requires that the gene expression is tuned in response to a yet-to-appear environmental cue. Moreover, regulatory crosstalk between cellular modules is ubiquitous in biology (Ravasz et al., 2002; Shen-Orr et al., 2002). One of the forms of specificity and crosstalk in regulation is between transcription factors and their recognition site on the DNA. Although each transcription factor binds its cognate regulatory site on the DNA, significant non-specific binding, and hence regulatory crosstalk, exists (Wunderlich and Mirny, 2009; Burger et al., 2010).

A particular form of crosstalk between two functional modules is when gene expression changes upon anticipation of an upcoming environmental shift. For example, in *Escherichia coli*, an increase in temperature elicits response to low oxygen conditions, mimicking how these two environments are sequentially encountered by the bacterium in the mammalian gastrointestinal tract (Tagkopoulos et al., 2008). Such anticipatory regulation was also demonstrated to occur between sugars lactose and maltose (Mitchell et al., 2009). Anticipatory regulation is seen in yeast too. Recent studies have demonstrated that yeast anticipates exhaustion of a primary carbon source, and thus, switches to the secondary source in the environment, even before the primary source is exhausted (New et al., 2014; Wang et al., 2015; Venturelli et al., 2015). Thus, fine-tuning of regulatory crosstalk between modules, leading to exhibition of anticipatory regulation, is done in accordance with the precise ecological niche of an organism.

Anticipatory gene regulation, as a strategy, is widespread in pathogens (Brunke and Hube, 2014). In *Mycobacterium*, the different two-component systems (TCSs) in the bacterium are wired so that activation of one leads to partial activation of the downstream TCSs (Agrawal et al., 2015). In *Salmonella*, flagella is assembled prior to assembly of a Type 3 Secretion System (T3SS) (Saini et al., 2010), and regulatory elements in the flagellar cascade are known to activate the SPI1-encoded T3SS genes (Chubiz et al., 2010). *Burkholderia*, in response to high cell density, anticipates stationary phase stress and triggers anticipatory response (Goo et al., 2012). Fungal pathogens have been shown to elicit anticipatory response for protection against attack from the immune system of the host (Pradhan et al., 2020).

Thus, repeated, temporal exposure of environmental cues may have led to evolution of regulatory crosstalk between cellular modules. Despite significant evidence that anticipatory gene expression provides an adaptive advantage to an organism, little is known about how anticipatory regulation can evolve in a population.

In this work, we ask the following question: if a population is exposed to two environmental signals, S1 and S2, sequentially and repeatedly, under what conditions can anticipatory regulation evolve as an adaptive strategy? To answer this question, we use a simple mathematical model to represent exposure of a population to two temporal stimuli [signal1 (S1) and signal2 (S2)] and identify the network and physiological parameters, which maximize fitness in the given conditions. Based on the inputs from our modeling results, we evolve *E. coli* under alternating exposure to a pentose sugar rhamnose (S1) and an oxidative stress molecule, paraquat (PQ) (S2) (see Supplementary Material 1 for more details).

Repeated, alternating exposure to S1 and S2 for ∼850 generations, leads to evolution of anticipatory regulation, where prior exposure to rhamnose provides an adaptive benefit when the population is shifted to paraquat. This benefit is observed only in lines, which were exposed to alternating S1 and S2; and is only seen when these cells are pre-exposed to rhamnose. Gene expression experiments demonstrate that one of the possible mechanisms of this adaptive benefit is partial activation of *soxS*, upon exposure to rhamnose. Our study thus demonstrates that, in controlled laboratory environment, anticipatory gene regulation can evolve in a short timeframe of a few hundred generations. Genome sequencing of the evolved lines reveals that anticipatory regulation can evolve *via* distinct molecular pathways, often involving mutations in global regulators.

## Materials and Methods

### Model System Description

**FIGURE 1:**
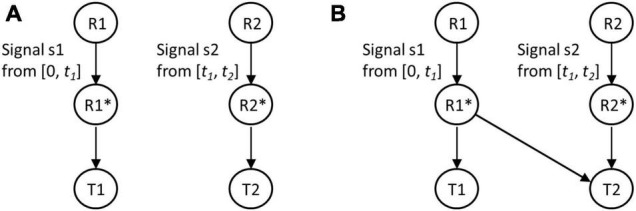
Regulatory designs without **(A)** and with **(B)** anticipatory regulation. In the prevailing environment, signal s1 is present from [0, *t1*] and signal s2 is present from [*t1*, *t2*]. In the first design, activated regulator R1* controls expression of target protein T1, and R2* controls expression of T2. In the second regulatory design, R1*, in addition to controlling expression of T1, also partially/fully controls expression of target protein T2.

Two regulatory modules and their possible interaction was studied as follows. A microorganism experiences an environmental signal S1. Upon sensing this signal, it modulates gene expression. As part of this response, a transcription factor R1 gets activated and triggers expression of a target protein T1. After time *t1*, S1 is replaced by another signal S2. In response to S2, the cell activates a transcription factor R2. The activated R2 then positively controls expression of the target protein T2, which confers an adaptive benefit in S2. The signal S2 persists for time *t2* (Figure 1).

In the first regulatory design, activated R1 is responsible for response to S1 (controlling expression of T1 only), and activated R2 controls expression of T2. We call this design a no-anticipatory regulation design (Figure 1A). In such a setting, when allowed to propagate in alternating S1 and S2 for a long time, the population will evolve and enhance fitness. In our mathematical framework, we solve for parameter values, which maximize fitness for the regulatory topology in Figure 1A.

On the other hand, in the design with anticipatory regulation, expression of T2 is controlled by active R1 as well as active R2 (Figure 1B). We solve for the values of these biochemical interaction that maximize fitness in this regulatory design. In particular, we are interested in identification of parameter values such that the fitness conferred by design in Figure 1B exceeds that by design in Figure 1A.

The equations dictating the synthesis and degradation rate of proteins in the cell, upon sensing signal S1 or S2 can be written as below.


(1) $$R1+\left[s1\right]↔R1^{*}$$

and,


(2) $$R2+\left[s2\right]↔R2^{*}$$

is represented as:


(3) $$k_{f1}\left[R1\right]\left[s1\right]↔k_{r1}\left[R1^{*}\right]$$

and


(4) $$k_{f2}\left[R2\right]\left[s2\right]↔k_{r2}\left[R2^{*}\right]$$

The dynamics of target protein T2 is represented as described in Equation 5. R2* is the transcription factor responsible for expression of T2, *Km2* is the Michaelis–Menten kinetic constant for T2 production, *b*_2_ is the maximum production rate of T2, and *k_*d*2_* is the combined degradation and dilution rate of T2 in the cell (Alon, 2006). The parameters *k*_f1_ (*k*_f2_) and *k*_r1_ (*k*_r2_) represent the rate constant of association and disassociation between the regulator R1 (R2) and the signal s1 (s2), respectively. We assume that expression of R1 and R2 is not contingent on the presence or absence of signals (but their activation is).


(5) $$\frac{dT2}{dt}=b_{2}\frac{R2^{*}}{R2^{*}+K_{m2}}-k_{d2}T2$$

However, in the case when anticipatory regulation controls gene expression (Figure 1B), the dynamics of T2 in the cell can be quantified as:


(6) $$\frac{dT2}{dt}=b_{1}\frac{R1^{*}}{R1^{*}+K_{m12}}+b_{2}\frac{R2^{*}}{R2^{*}+K_{m2}}-k_{d2}T2$$

Where,


$$K_{m}=\frac{k\_off}{k\_on}$$

for each transcription factor-DNA interaction, and *b*_1_ (*b*_2_) is the maximal strength of the *T2* promoter when driven by the activated transcription factor R1 (R2). In this manner, given the biochemical parameters of a network, we can quantify the dynamics of protein expression in a cell.

### Cost-Benefit Framework

From the time-course data of protein expression, we use a cost-benefit model to understand the cellular fitness, as a result of the gene expression dynamics (Dekel and Alon, 2005; Zaslaver et al., 2006b; Roselius et al., 2014; Fritz et al., 2019). In this formulation, the benefit conferred to the cell by the target protein T2 is given by the following expression:


(7) $$Benefit,E=E_{max}\frac{\left[T2\right]}{\left[T2\right]+K_{mT2}}$$

In addition, protein production is known to have a cost associated with it (Kafri et al., 2016). This cost is a linear function of the amount of protein being synthesized.


(8) $$Cost,c=c_{o}\left[T2\right]$$

The overall fitness of a cell is given by,


(9) Fitness,f=E-c

Given a set of network topology and the biochemical parameters associated with it, this framework allows us to compute the benefit of production of T2 and track this quantity with time. In the context of this work, we compute the fitness conferred by T2 in the time window [*t1, t2*] and the total cost associated with synthesis of protein T2 in the window [*t0*, *t2*].

### Solving for Optimal Fitness

We assume that the parameters associated with changing the regulatory patterns of gene expression (like, *Km* and *b*) are evolvable on a time scale faster than the time needed to change parameters associated with protein property (like, enzyme activity, degradation rate) (Babu et al., 2004; Wray, 2007; Wittkopp and Kalay, 2011; Rubinstein and de Souza, 2013). As a result, we let the parameters, which control regulation evolve, while keeping the others constant. Moreover, in our simulations, the value of each parameters is constrained by the thermodynamics associated with biological processes (Supplementary Material 2). The range chosen for each parameter represents the biologically permissible window in which they can take a value (Ozbudak et al., 2002; Rosenfeld et al., 2005, 2007; Mitrophanov and Groisman, 2008; Sneppen et al., 2010).

Given the above, we introduce “mutations” (change parameters) in the network and for each of the two regulatory designs (with and without anticipatory regulation, as defined above), find the parameter set which optimizes fitness. We are particularly interested in finding whether fitness is optimized *via* topology in Figures 1A or B.

All simulations were performed in Matlab 7.0.

### Strain Used

*Escherichia coli* K12 MG1655 (ATCC 47076) (F^–^ lambda^–^) was used in this study. The *soxS* mutant was generated using the primers 5′-CCCCAACAGATGAA TTAACGAACTGAACACTGAAA AGAGG GTGTAGGCTGG AGCTGCTTC-3′ and 5′-GAGCAATTACCCGCGCGGGAGT TAA CGCGCGGGCAATAAACATATGAATATCCTCCTTA-3′ from the parent strain, as per the method described by Datsenko and Wanner (2000).

### Media and Reagents

M9 glycerol medium contained 0.2% glycerol unless otherwise stated. M9 salts, trace elements and casamino acids were prepared in concentrated stocks. Stock of a 5× M9 salts solution consisted of 64 g/L Na_2_HPO_4_.7H_2_O, 15 g/L KH_2_PO_4_, 2.5 g/L NaCl, and 5 g/L NH_4_Cl dissolved in Milli-Q filtered water. Casamino acids were prepared as 10× solution and were used at a final concentration of 0.05% (w/v) in the growth media. MgSO_4_ and CaCl_2_ were prepared at 1M stock solutions each. Stocks of rhamnose and paraquat was prepared at 20% and 5 mM, respectively, and sterilized by filtration through 0.22 μm filters.

### Laboratory Evolution Experiment

Rhamnose (S1) and paraquat (S2) at a concentration of 0.2% and 40 μM, respectively, were used as the two stimuli. The evolution experiment was carried out in three replicate lines serially diluted 1:100 in alternating conditions of rhamnose and paraquat every 12 h, as described above. Control lines (in triplicate) were evolved in either 0.2% rhamnose only or 40 μM paraquat only. The control experiment were both the stimuli were added together did not grow beyond four dilutions, hence was dropped out of the experiment.

All cultures were grown at 37°C and 250 rpm. Sub-cultures in fresh media were done every 12 h (1:100) to ensure that cells did not enter stationary phase (Navarro Llorens et al., 2010). The evolution experiment was carried out for a total of 850 generations. Freezer stocks of intermediate time points were made in the experiment. Analysis of the stocks at 300 and 600 generations are presented in this work. Starting from the ancestor, three independent lines were evolved for each environmental condition.

### Analysis of Evolved Lines for Anticipatory Regulation

All evolved lines and the ancestor were revived from freezer stocks into 2 ml LB and incubated for 12 h with shaking at 250 rpm at 37°C. The cultures were then sub-cultured 1:100 in 2 ml M9 medium, containing 0.2% glycerol as the carbon source, for 12 h. From each tube, cells were then sub-cultured 1:100 in 2 ml M9 glycerol media (a) with and (b) without 0.2% rhamnose, and allowed to grow for 12 h at 250 rpm at 37°C. After growth for 12 h, all lines were sub-cultured to the same initial OD (0.1) into 2 ml M9 glycerol with PQ (40 μM). A volume of 150 μL of these cultures were transferred to a 96-well clear flat-bottom microplate (Costar) in triplicates. The cultures were grown at 37°C in a microplate reader (Tecan Infinite M200 Pro), until they reached stationary phase. OD600 readings were taken every 30 min with 10 min of orbital shaking at 5 mm amplitude before the readings. A gas permeable *Breathe-Easy* (Sigma-Aldrich) sealing membrane was used to seal the 96-well plates. Growth rate (Malthusian parameter, *r*) was calculated from the time to reach an OD 1.0, assuming exponential growth in this duration. This growth rate is presented in the growth rates in Figures 4D, 5.

**FIGURE 2:**
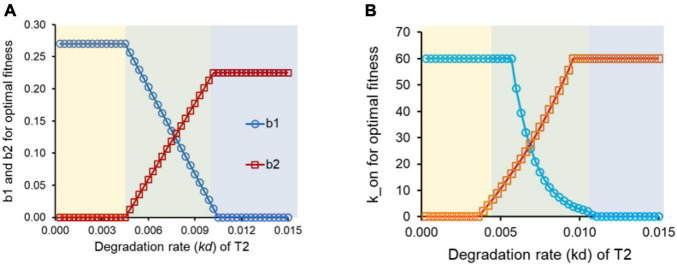
**(A)** For intermediate values of the degradation rate *kd* for target protein, T2, conditioning is the optimal response. **(B)** For intermediate values of the degradation rate *kd* for target protein, T2, conditioning is the optimal response.

**FIGURE 3:**
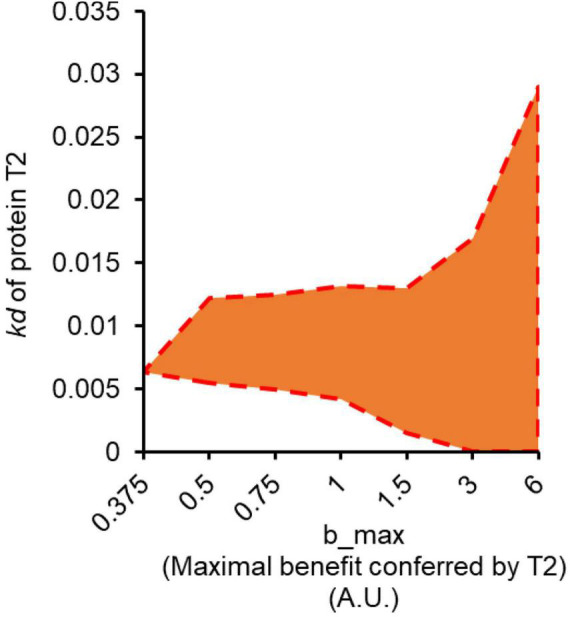
Anticipatory regulation emerges as optimal solution as maximum benefit conferred by T2 increases. The shaded region in orange represents the range of the values of *kd* for protein T2, for which anticipatory regulation is the optimal regulatory design, at a given value of *b*_*max*_.

**FIGURE 4:**
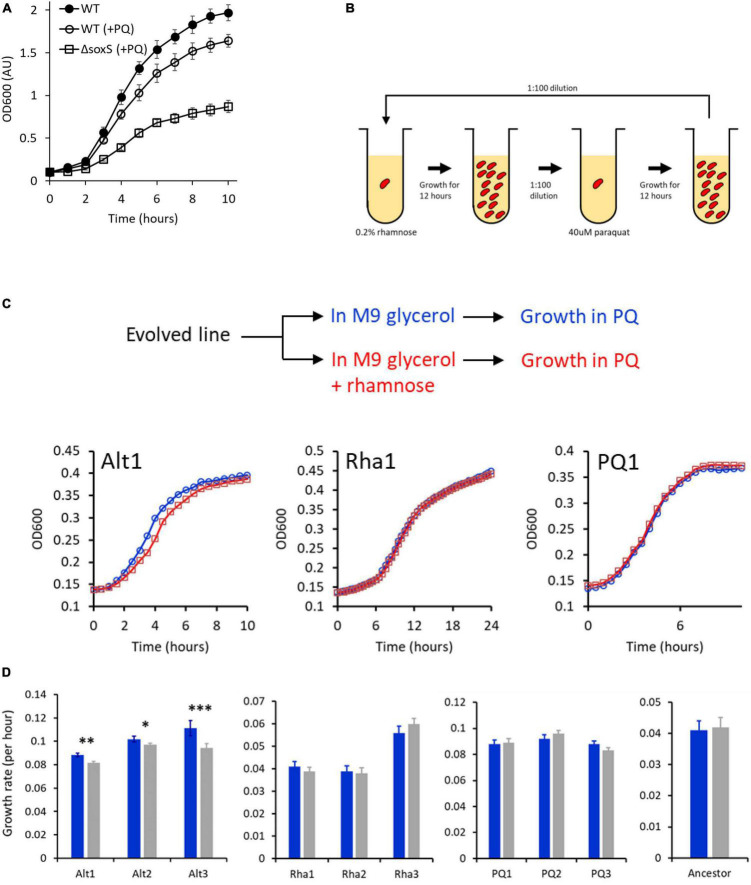
**(A)** *Escherichia coli* exhibits a growth defect in M9 glycerol media, when grown in the presence of 40 μM PQ. **(B)** Experimental plan for the rhamnose-PQ alternating evolution. Cells were exposed to rhamnose and then PQ for 6–7 generations for a total of 850 generations. **(C)** Anticipatory regulation evolves in rhamnose-PQ alternating lines. Cells from rhamnose-PQ evolved line (line Alt1) (left), rhamnose-only evolved line (line Rha1) (middle), and PQ-only evolved line (PQ1) (right) were grown in M9 minimal glycerol media with (blue) or without (red) rhamnose. The cells were transferred 1:100 to M9 glycerol media with 40 μM PQ, and their growth kinetics observed. (lines 1 from alternating rhamnose-PQ; only rhamnose; and only PQ.) **(D)** Anticipatory regulation provides a growth advantage in all rhamnose-PQ alternating evolved lines. Growth rates of the evolved lines in M9 glycerol media containing 40 μM PQ, when transferred from M9 glycerol media with (blue) or without (gray) 0.2% rhamnose. (^***^p-value < 0.01; ^**^p-value < 0.02; *p-value < 0.15; two-tailed, paired *t*-test).

**FIGURE 5:**
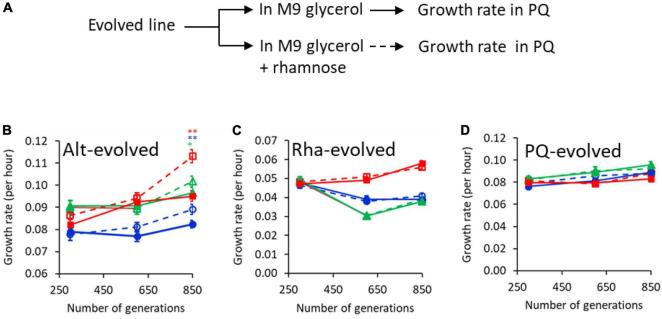
Growth advantage because of evolution of anticipatory regulation takes place around and after 600 generations into the experiment. **(A)** Experimental design. Evolved lines were grown in M9 glycerol and M9 glycerol containing rhamnose separately. The two cultures were transferred to M9 media containing PQ, and growth kinetics monitored. Growth rate of lines 1 (blue), 2 (green), and 3 (red) in rhamnose-PQ alternating conditions **(B)**, rhamnose-only **(C)**, and PQ only **(D)**. Solid and dashed lines represent growth rates of the lines, when transferred from M9 glycerol medium without or with rhamnose, respectively. (* represents *p*-value < 0.1; ** represents *p*-value < 0.0.1 in two-tailed, paired *t*-test).

### Gene Expression Measurements

The evolved lines and wild-type were transformed with *soxS*-GFP, a plasmid-based promoter fusion from Thermo Scientific *E. coli* promoter collection (PEC3877) (Zaslaver et al., 2006a). For estimating the promoter activity, cells were revived from freezer stocks from the −80°C deep freezer into 2 ml LB containing 40 μg/ml Kanamycin and incubated for 12 h at 37°C. The saturated cultures were then sub-cultured 1:100 in 2 ml M9 glycerol medium for 12 h. All lines were then sub-cultured 1:100 into fresh M9 glycerol medium without/with 0.2% rhamnose and allowed to grow for 12 h at 37°C. After this period of growth, 150 μL of these cultures was transferred to a 96-well Black clear flat-bottom microplate (Costar) and Fluorescence (488/525 nm) and OD600 readings taken using a microplate reader.

For *soxS*-GFP expression in presence of paraquat, cells were transferred at a 1:100 dilution from M9 glycerol to M9 glycerol with/without 0.2% rhamnose. The culture were then allowed to grow for 12 h at 37°C. The resulting cultures were diluted to an initial OD600 of 0.1 in 2 ml M9 glycerol media with 40 μM PQ. A volume of 150 μL of these cultures were transferred to a 96-well Black clear flat-bottom microplate in triplicates. The cultures were grown at 37°C in a microplate reader, until they reached stationary phase. Fluorescence (488/525 nm) and OD600 readings were taken every 30 min with 10 min of orbital shaking at 5 mm amplitude before the readings. A gas permeable *Breathe-Easy* (Sigma-Aldrich) sealing membrane was used to seal the 96-well plates. In this assay, the cumulative promoter strength over a period of time is measured.

A two-tailed *t*-test was performed to compare two sets of data. The *p*-value corresponding to four degrees of freedom was obtained using the calculated *t*-statistic for each set.

### Whole Genome Sequencing and Variant Calling

#### Sample Preparation and Sequencing

Genomic DNA of ancestral and evolved lines were isolated following standard zymolyase-based protocol using the kit from FAVORGEN Biotech Corporation. DNA concentrations and quality were measured using a Nano-spectrophotometer from Eppendorf and by gel electrophoresis. Samples were paired-end sequenced on an Illumina HiSeq, with an average read length of 150 bp. Each sample had a minimum coverage of 100×.

#### Mapping and Variant Calling

A cloud-based web interface system Galaxy^1^ was used to perform all sequence data analysis. Illumina paired-end reads were uploaded into the server. The quality of the reads was assessed using FastQC (Version 0.72) (Andrews, n. d.). The reads were mapped to *E. coli* str. K-12 substr. MG1655 genome (GCA_000005845.2) and variant calling was performed using the automated tool Snippy (Version 3.2) (Seemann, 2015), according to the recommendations for evaluating single nucleotide variant calling (Olson et al., 2015). Variants present in the ancestral strain were filtered out manually. Finally, all remaining indels and SNPs were verified using intensive manual curation.

## Results

### Anticipatory Regulation Is an Adaptive Response for Intermediate Values of Degradation Rates of the Protein T2

We simulate the networks in Figures 1A,B for a set of parameter values, chosen from a window typically representing each biochemical interaction in a cell (Ozbudak et al., 2002; Rosenfeld et al., 2005, 2007; Suel et al., 2006; Mitrophanov and Groisman, 2008; Sneppen et al., 2010; Prajapat et al., 2016). For a given value of *kd* (degradation + dilution rate constant for protein T2), we find the regulatory design in Figure 1, which leads to a maximal fitness.

Maximal fitness is obtained *via* three distinct regulatory logics, depending on the degradation rate of the protein T2 (Figure 2A). These region corresponding to the three designs is as shown in the bands in Figures 2A,B (yellow, green, and blue). For small values of the *kd* of T2, the maximal fitness is achieved when control of T2 is entirely under the regulator R1. That is, R2 does not play any role in controlling expression of T2. This can be understood as follows. Expression of T2 by R1 leads to T2 production even before signal S2 is present in the environment. As a result, the T2 so produced does not confer any advantage to the cell. Thus, this preemptive production of T2 does not give any benefit to the cell, but does come at an additional cost associated with protein production in the cell. This cost, however, is offset by the enhanced T2 levels inside the cell (and consequently the benefit conferred to the cell), from the time when signal S2 is introduced to the system. When T2 is stable, the steady state T2 in the cell is high, and as a result, the fitness of the cell at the moment S2 is introduced to the system is also high.

At the other extreme, when the *kd* of T2 is high, the regulatory design that optimizes cellular fitness is one where control of expression of T2 is not by R1, but is instead entirely by R2. In such a context, the energetic cost of T2 production in the “futile” time-period [0, *t1*] prior to introduction of signal B, because of the high protein turnover, is high. In such a scenario, the cellular adaptive strategy is control of each target protein by its cognate regulatory partner.

However, at intermediate values of the protein T2 degradation rate constant, for maximal cellular fitness, both *b*_1_ and *b*_2_ are non-zero. This window of the values of the degradation rate constant of T2 represents the scenario where conditioning is the adaptive response in cellular functioning. Such a set up identifies conditions for a distributed regulatory design for control of gene expression (Saini et al., 2010). However, anticipatory regulation may or may not be accessible from a given starting point on the landscape (Blount et al., 2018; Brajesh et al., 2019; Fragata et al., 2019).

Changes in regulatory design can also be incorporated by changing the binding affinity of the transcription factor with the promoter region of the DNA. In the context of the model, we change the parameter k_on, which represents the affinity of the DNA for the transcription factor. Similar to the results obtained above, anticipatory regulation outperforms the regulatory design in Figure 1A for intermediate values of the degradation rate of protein T2, when we permit k_on to vary for protein T2 (Figure 2B).

### Can Anticipatory Regulation Be Evolved in a Laboratory Set-up?

We use the model to text the conditions in which conditioning would be the optimum response (compared to non-conditioning response). When we scan the two parameter region defined by *E*_*max*_ (maximum benefit conferred by T2) and *kd* of protein T2, we note that as *E*_*max*_ increases, conditioning emerges as the optimal solution for an increasing window of the parameter range *kd* for protein T2 (Figure 3).

From these results, we predict that anticipatory regulation is most likely to evolve if the target protein confers a large fitness advantage to the cell. This is perhaps the single most important criteria for anticipatory regulation to emerge as an outcome of an evolutionary experiment. In other words, anticipatory regulation is more likely to be the optimal regulatory design when absence of T2 causes a significant fitness cost to the cell. Therefore, for experimental evolution of anticipatory regulation, we choose a cellular stress as S2.

### Evolution of Anticipatory Regulation in *Escherichia coli*

From our modeling exercise, we note that the key parameter for evolution of anticipatory regulation is cost associated with not initiating a rapid response to signal S2. In view of these inputs, we chose a pair of environmental signals, which meet this criteria. We chose the pentose sugar rhamnose as signal S1 and an oxidative stress molecule, paraquat (PQ) as signal S2 (see Supplementary Material 1 for more details of the two systems). To the best of our knowledge, the two systems are not linked by any direct transcriptional regulation. A stress of 40 μM PQ induces a growth defect in *E. coli* (Figure 4A). Thus, any anticipated expression of the SoxRS regulon is likely to provide a large adaptive benefit to the individual. With this premise, we performed an evolutionary experiment where the cells were exposed to S1 and S2 alternatively for a total of about 850 generations (Figure 4B; see section “Materials and Methods”).

In such a context, anticipatory regulation can be said to have evolved when the following two conditions are met. First, after alternating exposure to rhamnose and PQ, pre-exposure to rhamnose confers a growth advantage when the cells are exposed to PQ. Second, that this advantage should only be present in the lines evolved in an environment of alternating rhamnose and PQ.

In Figure 4C, growth kinetics of one of the lines from the three experimental conditions are shown. Cells evolved in alternating rhamnose-PQ were pre-grown in (a) glycerol and (b) glycerol and rhamnose. The two cultures were transferred to a M9 glycerol media containing PQ, and the growth kinetics compared. As shown in Figure 4C, the cells pre-exposed to rhamnose exhibit a growth advantage in PQ environment. This advantage is absent in the lines evolved in rhamnose-only, or PQ-only. In Figure 4D, the growth rate for all three lines in each of the three environments is shown. Among all the lines, only rhamnose-PQ alternating evolved lines show a growth advantage, when pre-exposed to rhamnose. The relative amounts of the advantage vary between the three lines. As expected, the rhamnose-evolved lines show a considerably lower growth rate as compared to the PQ-evolved and the rhamnose-PQ alternating evolved lines.

To test the kinetics of the growth advantage during the course of our evolution experiment, two intermediate time-points (generation 300 and 600) in the evolutionary experiment were also checked for their growth rate. As shown in Figure 5, no growth advantage due to anticipatory regulation was observed at 300 generations. By 600 generations, anticipatory regulation was observed in two of the three rhamnose-PQ alternating lines. This trend was exaggerated by generations 850. The growth advantage, associated with a prior exposure to rhamnose, is not observed in rhamnose-only and PQ-only evolved lines. Interestingly, in two of the three rhamnose-evolved lines, fitness decreases in the PQ environment, indicating trade-off (Cooper and Lenski, 2000; Litchman et al., 2015; Ferenci, 2016).

### Evolved Lines Lead to Faster Induction of SoxS

**FIGURE 6:**
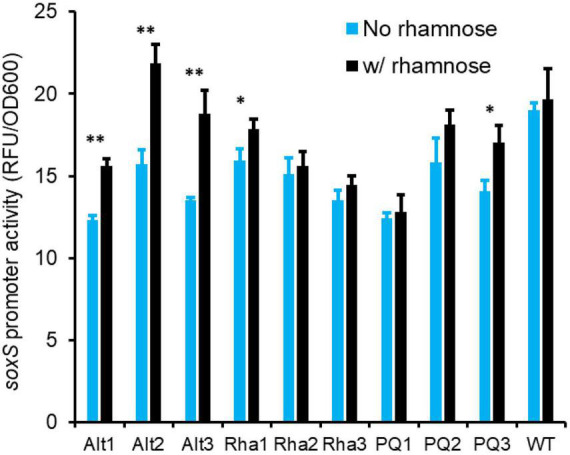
Lines evolved with alternating exposure to rhamnose and paraquat (Alt1, Alt2, and Alt3) exhibit a higher expression from the *soxS* promoter after growth for 8 h in the presence of rhamnose. This rhamnose-dependent activation of the *soxS* promoter is absent in the ancestor (WT), in rhamnose-evolved lines (Rha1, Rha2, and Rha3), and in paraquat-evolved lines (PQ1, PQ2, and PQ3) (^**^ indicates *p*-value < 0.002, * indicates *p*-value < 0.02, two-tailed, paired *t*-test).

SoxS is one of the key proteins responsible for cellular oxidative stress response (Seo et al., 2015). To test if induction of SoxS differs in the evolved and ancestral lines, when the cells are exposed to rhamnose, we grew *E. coli* in M9 glycerol media in the absence and presence of 0.2% rhamnose. As shown in Figure 6, in all three lines evolved with alternating exposure to rhamnose and paraquat, *soxS* promoter is activated in presence of 0.2% rhamnose. This rhamnose-dependent activation of the *soxS* promoter is absent in the lines evolved in rhamnose only, or the lines evolved in paraquat only. In addition, rhamnose-dependent activation of the *soxS* promoter is absent in the ancestor strain.

When lines Alt1, Alt2, and Alt3 were transferred from M9 glycerol media with and without rhamnose to M9 glycerol media containing paraquat, cells transferred from environments with rhamnose exhibited a faster induction of the *soxS* promoter (Supplementary Material 3).

This data demonstrates that in the lines evolved in alternating rhamnose and paraquat, anticipatory regulation evolves. This anticipatory regulation leads to a growth phenotype. Our results here demonstrate that one of the manifestations of evolution of anticipatory regulation is partial induction of the *soxS* promoter, when cells are grown in rhamnose. This partial induction leads to a faster transition to the ON state, when cells transition to an environment containing paraquat.

### Molecular Basis of Anticipatory Regulation

Mutations in the evolved lines were identified from the genome sequencing results. A complete list of the mutations in the evolved lines is in Supplementary Material 4. All three lines had SNPs in *glpK*. Glycerol kinase (GlpK) is involved in the utilization of the glycerol moiety of phospholipids and triglycerides after their breakdown into usable forms (Lin et al., 1962; Zwaig and Lin, 1966; Zwaig et al., 1970; Thorner and Paulus, 1973; Herring et al., 2006; Joyce et al., 2006). In several adaptive evolution experiments, growth on glycerol has led to mutations in *glpK* coding region (Honisch et al., 2004; Herring et al., 2006; Applebee et al., 2008, 2011; Cheng et al., 2014). Our studies report three novel *glpK* alleles, which improve growth rate in minimal media containing glycerol.

Additionally, each of the three lines acquired other mutations. Adenylate cyclase (encoded by *cyaA*) catalyzes synthesis of cyclic AMP (cAMP). Mutations in cyaA has also been reported to confer high hydrostatic pressure resistance and increased antibiotic resistance in *E. coli* (Nishino et al., 2008; Gayan et al., 2017). Moreover, because of cyclic AMP receptor protein (CRP)-cAMP complex has a global affect on gene regulation, the gene expression patterns in the cell in a *cya* mutant are qualitatively different, compared to the wild-type (Delaney, 1990). A mutation identified in one of the lines (Alt3) is a SNP in the RNA component of the small subunit (30S subunit) of the *E. coli* ribosome. The 16S rRNA is targeted by a number of antibiotic agents (Thompson and Hearst, 1983; Stern et al., 1989; Brimacombe, 1992; Schneider et al., 2003). As a result of this mutation, the proteome profile in the cell is known to be changed, affecting expression levels of more than a hundred proteins. Line Alt2 acquired a mutation in *rpoS*, which regulates transcription of a large number of genes related to sugar metabolism and polyamine metabolism in response to cellular stresses. It also has important roles in nucleic acid synthesis, modification, and turnover (Maciag et al., 2011); and response to oxidative stress (Soo and Wood, 2013). Polymorphisms in *rpoS* that affect fitness across different environments are commonly found in *E. coli* (Notley-McRobb et al., 2002; Zinser and Kolter, 2004; King et al., 2006).

Another class of mutations was identified in line Alt1. These include a mutation in *potA*, which impacts susceptibility to oxidative stresses (Tabor and Tabor, 1964; Abraham, 1968; Mitsui et al., 1984; Schuber, 1989; Chattopadhyay et al., 2003). Line Alt1 also acquired a mutation in TrkH, a potassium ion transporter (Dosch et al., 1991; Schlosser et al., 1995). A mutation in *trkH* is often associated with aminoglycoside resistance in bacteria (Lazar et al., 2013; Apjok et al., 2019). It also plays important roles in osmotic response, modulation of membrane potential, pH maintenance, and overall fitness in a variety of bacterial species (Castaneda-Garcia et al., 2011; Ochrombel et al., 2011; Quintero-Yanes et al., 2019).

Overall, while the mechanistic explanation for the observed anticipatory regulation, and the corresponding increase in fitness is difficult to understand fully from the sequencing results, our results demonstrate how small mutations in global regulators of a cell can bring about fine-tuning of gene expression which leads to changes in timing and strength of gene expression, contributing to cellular fitness.

## Discussion and Conclusion

Ecological niches often have cyclical cues – e.g., temperature increase followed by decrease in oxygen availability upon ingestion; nutritional cues are spatial in nature in the small intestine. Presumably, repeated exposure to these cues leads to evolution of anticipatory regulation. However, the timeline, mechanisms *via* which anticipatory regulation evolves is not known. This work is a first attempt toward this question.

Repeated exposure to two cues (rhamnose and PQ) lead to evolution of anticipatory regulation. While the phenomenology is reported in our study, the mechanistic details of this phenomenon is not identified. Our future work is directed toward answering this question. Our results suggest the possibility that *via* mutations in global regulators, the gene expression pattern of the cell is changed so that prior exposure to rhamnose leads to a fitness advantage when cells are exposed to oxidative stress. Interestingly, this phenomenon is observed without mutations in the *rhaSR* region or the *soxSR* region of the *E. coli* genome. The precise mechanistic details of the rhamnose-dependent upregulation of *soxS* remains unknown. Several possibilities exist in this regard. Expression of rhamnose utilization genes could lead to a pleiotropic effect on gene expression, leading to partial expression of genes involved in tackling oxidative stress. Alternatively, change in global regulators (as indicated in the mutations in Supplementary Table 1), and an altered metabolic state (due to presence of rhamnose) leads to upregulation of *soxS* and presumably, its regulon. Gene regulation can also be incorporated *via* post-transcription, translation, and/or post-translation mechanisms, while our model only considers transcriptional rewiring. Post-transcriptional modes of regulation can, structurally, be added to the modeling details. However, since most biochemical parameters are unknown, the utility of inputs from these models will likely be limited. On the other hand, our model, albeit simple, predicts the effect that use of a stress as a signal leads to a greater chance of anticipatory regulation to evolve.

In this work, we present experimental evidence of evolution of anticipatory regulation in bacteria. We demonstrate that by evolving *E. coli* in alternating environments of rhamnose and PQ, anticipatory regulation evolves, where prior exposure to rhamnose enhances fitness of the population in PQ. In our experimental set-up this adaptation evolves in the time scale of a few hundred generations. Anticipatory regulation involves mutations in global regulators (like *cya*, *rpoS*, ribosomal subunit), which likely lead to large-scale changes in transcriptional and translational profile of the cell. Mutations which confer a direct fitness advantage in the evolved environment are also reported (e.g., all three lines have a SNP is *glpK*). A previous attempt to evolve anticipatory regulation in yeast was made by choice of two stresses as environmental cues. However, disentangling the effect of the two stresses made the analysis of such a system challenging (Dhar et al., 2013). Overall, our study demonstrates clearly, that anticipatory regulation of gene regulation can evolve in a few hundred generations. Understanding this largely unexplored mode of regulation can help us understand the arrangement of global regulatory patterns in bacteria.

## Data Availability Statement

The datasets presented in this study can be found in online repositories. The names of the repository/repositories and accession number(s) can be found in the article/Supplementary Material.

## Author Contributions

AnM carried out the evolution experiment, performed gene expression measurements, sequence analysis, and wrote the manuscript. PV and AkM performed the simulations and analyzed the data. SS and AnM conceived the study and wrote the manuscript. All authors gave final approval for publication and agreed to be held accountable for the work performed therein.

## Conflict of Interest

The authors declare that the research was conducted in the absence of any commercial or financial relationships that could be construed as a potential conflict of interest.

## Publisher’s Note

All claims expressed in this article are solely those of the authors and do not necessarily represent those of their affiliated organizations, or those of the publisher, the editors and the reviewers. Any product that may be evaluated in this article, or claim that may be made by its manufacturer, is not guaranteed or endorsed by the publisher.

## Footnotes

1
https://usegalaxy.eu/

## References

[B1] Abraham K. A. (1968). Studies on DNA-dependent RNA polymerase from *Escherichia coli*. 1. The mechanism of polyamine induced stimulation of enzyme activity. *Eur. J. Biochem.* 5 143–146. 10.1111/j.1432-1033.1968.tb00348.x 4873311

[B2] Agrawal R. Pandey A. Rajankar M. P. Dixit N. M. Saini D. K. (2015). The two-component signalling networks of Mycobacterium tuberculosis display extensive cross-talk *in vitro*. *Biochem. J.* 469 121–134. 10.1042/BJ20150268 25929189

[B3] Alon U. (2006). *An Introduction to Systems Biology: Design Principles of Biological Circuits.* Boca Raton: Chapman and Hall/CRC.

[B4] Andrews S. (n. d.). *FastQC A Quality Control tool for High Throughput Sequence Data.* Available Online at: http://www.bioinformatics.babraham.ac.uk/projects/fastqc/ (accessed August 15, 2021).

[B5] Apjok G. Boross G. Nyerges A. Fekete G. Lazar V. Papp B. (2019). Limited evolutionary conservation of the phenotypic effects of antibiotic resistance mutations. *Mol. Biol. Evol.* 36 1601–1611. 10.1093/molbev/msz109 31058961 PMC6657729

[B6] Applebee M. K. Herrgard M. J. Palsson B. O. (2008). Impact of individual mutations on increased fitness in adaptively evolved strains of *Escherichia coli*. *J. Bacteriol.* 190 5087–5094. 10.1128/JB.01976-07 18487343 PMC2447027

[B7] Applebee M. K. Joyce A. R. Conrad T. M. Pettigrew D. W. Palsson B. O. (2011). Functional and metabolic effects of adaptive glycerol kinase (GLPK) mutants in *Escherichia coli*. *J. Biol. Chem.* 286 23150–23159. 10.1074/jbc.M110.195305 21550976 PMC3123082

[B8] Babu M. M. Luscombe N. M. Aravind L. Gerstein M. Teichmann S. A. (2004). Structure and evolution of transcriptional regulatory networks. *Curr. Opin. Struct. Biol.* 14 283–291. 15193307 10.1016/j.sbi.2004.05.004

[B9] Blount Z. D. Lenski R. E. Losos J. B. (2018). Contingency and determinism in evolution: replaying life’s tape. *Science* 362:eaam5979. 10.1126/science.aam5979 30409860

[B10] Brajesh R. G. Dutta D. Saini S. (2019). Distribution of fitness effects of mutations obtained from a simple genetic regulatory network model. *Sci. Rep.* 9:9842. 10.1038/s41598-019-46401-7 31285500 PMC6614479

[B11] Brimacombe R. (1992). Structure-function correlations (and discrepancies) in the 16S ribosomal RNA from *Escherichia coli*. *Biochimie* 74 319–326. 10.1016/0300-9084(92)90109-r 1379076

[B12] Brunke S. Hube B. (2014). Adaptive prediction as a strategy in microbial infections. *PLoS Pathog.* 10:e1004356. 10.1371/journal.ppat.1004356 25275642 PMC4183746

[B13] Burger A. Walczak A. M. Wolynes P. G. (2010). Abduction and asylum in the lives of transcription factors. *Proc. Natl. Acad. Sci. U. S. A.* 107 4016–4021. 10.1073/pnas.0915138107 20160109 PMC2840107

[B14] Castaneda-Garcia A. Do T. T. Blazquez J. (2011). The K+ uptake regulator TrkA controls membrane potential, pH homeostasis and multidrug susceptibility in *Mycobacterium smegmatis*. *J. Antimicrob. Chemother.* 66 1489–1498. 10.1093/jac/dkr165 21613307

[B15] Chattopadhyay M. K. Tabor C. W. Tabor H. (2003). Polyamines protect *Escherichia coli* cells from the toxic effect of oxygen. *Proc. Natl. Acad. Sci. U. S. A.* 100 2261–2265. 10.1073/pnas.2627990100 12591940 PMC151328

[B16] Cheng K. K. Lee B. S. Masuda T. Ito T. Ikeda K. Hirayama A. (2014). Global metabolic network reorganization by adaptive mutations allows fast growth of *Escherichia coli* on glycerol. *Nat. Commun.* 5:3233. 10.1038/ncomms4233 24481126

[B17] Chubiz J. E. C. Golubeva Y. A. Lin D. X. Miller L. D. Slauch J. M. (2010). FliZ Regulates Expression of the *Salmonella* Pathogenicity Island 1 Invasion Locus by Controlling HilD Protein Activity in *Salmonella enterica* Serovar Typhimurium. *J. Bacteriol.* 192 6261–6270. 10.1128/JB.00635-10 20889744 PMC2981222

[B18] Cooper V. S. Lenski R. E. (2000). The population genetics of ecological specialization in evolving *Escherichia coli* populations. *Nature* 407 736–739. 10.1038/35037572 11048718

[B19] Datsenko K. A. Wanner B. L. (2000). One-step inactivation of chromosomal genes in *Escherichia coli* K-12 using PCR products. *Proc. Natl. Acad. Sci. U. S. A.* 97 6640–6645. 10.1073/pnas.120163297 10829079 PMC18686

[B20] Dekel E. Alon U. (2005). Optimality and evolutionary tuning of the expression level of a protein. *Nature* 436 588–592. 10.1038/nature03842 16049495

[B21] Delaney J. M. (1990). A cya deletion mutant of *Escherichia coli* develops thermotolerance but does not exhibit a heat-shock response. *Genet. Res.* 55 1–6. 10.1017/s001667230002512x 2180785

[B22] Dhar R. Sagesser R. Weikert C. Wagner A. (2013). Yeast adapts to a changing stressful environment by evolving cross-protection and anticipatory gene regulation. *Mol. Biol. Evol.* 30 573–588. 10.1093/molbev/mss253 23125229

[B23] Dosch D. C. Helmer G. L. Sutton S. H. Salvacion F. F. Epstein W. (1991). Genetic analysis of potassium transport loci in *Escherichia coli*: evidence for three constitutive systems mediating uptake potassium. *J. Bacteriol.* 173 687–696. 10.1128/jb.173.2.687-696.1991 1987159 PMC207060

[B24] Ferenci T. (2016). Trade-off mechanisms shaping the diversity of bacteria. *Trends Microbiol.* 24 209–223. 10.1016/j.tim.2015.11.009 26705697

[B25] Fragata I. Blanckaert A. Dias Louro M. A. Liberles D. A. Bank C. (2019). Evolution in the light of fitness landscape theory. *Trends Ecol. Evol.* 34 69–82. 10.1016/j.tree.2018.10.009 30583805

[B26] Fritz G. Walker N. Gerland U. (2019). Heterogeneous timing of gene induction as a regulation strategy. *J. Mol. Biol.* 431 4760–4774. 10.1016/j.jmb.2019.05.020 31141707

[B27] Gayan E. Cambre A. Michiels C. W. Aertsen A. (2017). RpoS-independent evolution reveals the importance of attenuated cAMP/CRP regulation in high hydrostatic pressure resistance acquisition in E. coli. *Sci. Rep.* 7:8600. 10.1038/s41598-017-08958-z 28819154 PMC5561100

[B28] Goo E. Majerczyk C. D. An J. H. Chandler J. R. Seo Y. S. Ham H. (2012). Bacterial quorum sensing, cooperativity, and anticipation of stationary-phase stress. *Proc. Natl. Acad. Sci. U. S. A.* 109 19775–19780. 10.1073/pnas.1218092109 23150539 PMC3511722

[B29] Herring C. D. Raghunathan A. Honisch C. Patel T. Applebee M. K. Joyce A. R. (2006). Comparative genome sequencing of *Escherichia coli* allows observation of bacterial evolution on a laboratory timescale. *Nat. Genet.* 38 1406–1412. 10.1038/ng1906 17086184

[B30] Honisch C. Raghunathan A. Cantor C. R. Palsson B. O. Van Den Boom D. (2004). High-throughput mutation detection underlying adaptive evolution of *Escherichia coli*-K12. *Genome Res.* 14 2495–2502. 10.1101/gr.2977704 15574828 PMC534674

[B31] Johnson M. E. Hummer G. (2011). Nonspecific binding limits the number of proteins in a cell and shapes their interaction networks. *Proc. Natl. Acad. Sci. U. S. A.* 108 603–608. 10.1073/pnas.1010954108 21187424 PMC3021073

[B32] Joyce A. R. Reed J. L. White A. Edwards R. Osterman A. Baba T. (2006). Experimental and computational assessment of conditionally essential genes in *Escherichia coli*. *J. Bacteriol.* 188 8259–8271. 10.1128/JB.00740-06 17012394 PMC1698209

[B33] Kafri M. Metzl-Raz E. Jona G. Barkai N. (2016). The cost of protein production. *Cell Rep.* 14 22–31. 10.1016/j.celrep.2015.12.015 26725116 PMC4709330

[B34] King T. Seeto S. Ferenci T. (2006). Genotype-by-environment interactions influencing the emergence of rpoS mutations in *Escherichia coli* populations. *Genetics* 172 2071–2079. 10.1534/genetics.105.053892 16489226 PMC1456365

[B35] Lazar V. Pal Singh G. Spohn R. Nagy I. Horvath B. Hrtyan M. (2013). Bacterial evolution of antibiotic hypersensitivity. *Mol. Syst. Biol.* 9:700. 24169403 10.1038/msb.2013.57 PMC3817406

[B36] Lin E. C. Koch J. P. Chused T. M. Jorgensen S. E. (1962). Utilization of L-alpha-glycerophosphate by *Escherichia coli* without hydrolysis. *Proc. Natl. Acad. Sci. U. S. A.* 48 2145–2150. 10.1073/pnas.48.12.2145 13930693 PMC221136

[B37] Litchman E. Edwards K. F. Klausmeier C. A. (2015). Microbial resource utilization traits and trade-offs: implications for community structure, functioning, and biogeochemical impacts at present and in the future. *Front. Microbiol.* 6:254. 10.3389/fmicb.2015.00254 25904900 PMC4389539

[B38] Maciag A. Peano C. Pietrelli A. Egli T. De Bellis G. Landini P. (2011). *In vitro* transcription profiling of the sigmaS subunit of bacterial RNA polymerase: re-definition of the sigmaS regulon and identification of sigmaS-specific promoter sequence elements. *Nucleic Acids Res.* 39 5338–5355. 10.1093/nar/gkr129 21398637 PMC3141248

[B39] Mitchell A. Romano G. H. Groisman B. Yona A. Dekel E. Kupiec M. (2009). Adaptive prediction of environmental changes by microorganisms. *Nature* 460 220–224. 19536156 10.1038/nature08112

[B40] Mitrophanov A. Y. Groisman E. A. (2008). Positive feedback in cellular control systems. *Bioessays* 30 542–555. 18478531 10.1002/bies.20769 PMC2486260

[B41] Mitsui K. Ohnishi R. Hirose S. Igarashi K. (1984). Necessity of polyamines for maximum *in vivo* synthesis of beta beta’ subunits of RNA polymerase. *Biochem. Biophys. Res. Commun.* 123 528–534. 10.1016/0006-291x(84)90261-4 6385968

[B42] Navarro Llorens J. M. Tormo A. Martinez-Garcia E. (2010). Stationary phase in gram-negative bacteria. *FEMS Microbiol. Rev.* 34 476–495. 10.1111/j.1574-6976.2010.00213.x 20236330

[B43] New A. M. Cerulus B. Govers S. K. Perez-Samper G. Zhu B. Boogmans S. (2014). Different levels of catabolite repression optimize growth in stable and variable environments. *PLoS Biol.* 12:e1001764. 10.1371/journal.pbio.1001764 24453942 PMC3891604

[B44] Nishino K. Senda Y. Yamaguchi A. (2008). CRP regulator modulates multidrug resistance of *Escherichia coli* by repressing the mdtEF multidrug efflux genes. *J. Antibiot. (Tokyo)* 61 120–127. 10.1038/ja.2008.120 18503189

[B45] Notley-McRobb L. King T. Ferenci T. (2002). rpoS mutations and loss of general stress resistance in *Escherichia coli* populations as a consequence of conflict between competing stress responses. *J. Bacteriol.* 184 806–811. 10.1128/JB.184.3.806-811.2002 11790751 PMC139526

[B46] Ochrombel I. Ott L. Kramer R. Burkovski A. Marin K. (2011). Impact of improved potassium accumulation on pH homeostasis, membrane potential adjustment and survival of *Corynebacterium glutamicum*. *Biochim. Biophys. Acta* 1807 444–450. 10.1016/j.bbabio.2011.01.008 21295539

[B47] Olson N. D. Lund S. P. Colman R. E. Foster J. T. Sahl J. W. Schupp J. M. (2015). Best practices for evaluating single nucleotide variant calling methods for microbial genomics. *Front. Genet.* 6:235. 10.3389/fgene.2015.00235 26217378 PMC4493402

[B48] Ozbudak E. M. Thattai M. Kurtser I. Grossman A. D. Van Oudenaarden A. (2002). Regulation of noise in the expression of a single gene. *Nat. Genet.* 31 69–73. 11967532 10.1038/ng869

[B49] Pradhan A. Ma Q. De Assis L. J. Leaves I. Larcombe D. E. Rodriguez Rondon A. V. (2020). Anticipatory stress responses and immune evasion in fungal pathogens. *Trends Microbiol.* 29 416–427. 10.1016/j.tim.2020.09.010 33059975

[B50] Prajapat M. K. Jain K. Choudhury D. Raj N. Saini S. (2016). Revisiting demand rules for gene regulation. *Mol. Biosyst.* 12 421–430. 10.1039/c5mb00693g 26627179

[B51] Quintero-Yanes A. Monson R. E. Salmond G. P. C. (2019). Environmental potassium regulates bacterial flotation, antibiotic production and turgor pressure in Serratia through the TrkH transporter. *Environ. Microbiol.* 21 2499–2510. 10.1111/1462-2920.14637 31012245 PMC6617781

[B52] Ravasz E. Somera A. L. Mongru D. A. Oltvai Z. N. Barabasi A. L. (2002). Hierarchical organization of modularity in metabolic networks. *Science* 297 1551–1555. 10.1126/science.1073374 12202830

[B53] Roselius L. Langemann D. Muller J. Hense B. A. Filges S. Jahn D. (2014). Modelling and analysis of a gene-regulatory feed-forward loop with basal expression of the second regulator. *J. Theor. Biol.* 363 290–299. 10.1016/j.jtbi.2014.08.043 25193818

[B54] Rosenfeld N. Young J. W. Alon U. Swain P. S. Elowitz M. B. (2005). Gene regulation at the single-cell level. *Science* 307 1962–1965. 10.1126/science.1106914 15790856

[B55] Rosenfeld N. Young J. W. Alon U. Swain P. S. Elowitz M. B. (2007). Accurate prediction of gene feedback circuit behavior from component properties. *Mol. Syst. Biol.* 3:143. 10.1038/msb4100185 18004276 PMC2132446

[B56] Rubinstein M. de Souza F. S. (2013). Evolution of transcriptional enhancers and animal diversity. *Philos. Trans. R. Soc. Lond. B Biol. Sci.* 368:20130017. 10.1098/rstb.2013.0017 24218630 PMC3826491

[B57] Saini S. Slauch J. M. Aldridge P. D. Rao C. V. (2010). Role of cross talk in regulating the dynamic expression of the flagellar *Salmonella* pathogenicity island 1 and type 1 fimbrial genes. *J. Bacteriol.* 192 5767–5777. 10.1128/JB.00624-10 20833811 PMC2953706

[B58] Schlosser A. Meldorf M. Stumpe S. Bakker E. P. Epstein W. (1995). TrkH and its homolog, TrkG, determine the specificity and kinetics of cation transport by the Trk system of *Escherichia coli*. *J. Bacteriol.* 177 1908–1910. 7896723 10.1128/jb.177.7.1908-1910.1995 PMC176828

[B59] Schneider D. A. Ross W. Gourse R. L. (2003). Control of rRNA expression in *Escherichia coli*. *Curr. Opin. Microbiol.* 6 151–156. 10.1016/s1369-5274(03)00038-9 12732305

[B60] Schuber F. (1989). Influence of polyamines on membrane functions. *Biochem. J.* 260 1–10. 10.1042/bj2600001 2673211 PMC1138618

[B61] Seemann T. (2015). *Snippy: Fast Bacterial Variant Calling From NGS Reads.* Available Online at: https://github.com/tseemann/snippy (accessed August 15, 2021).

[B62] Seo S. W. Kim D. Szubin R. Palsson B. O. (2015). Genome-wide reconstruction of OxyR and SoxRS transcriptional regulatory networks under oxidative stress in *Escherichia coli* K-12 MG1655. *Cell Rep.* 12 1289–1299. 10.1016/j.celrep.2015.07.043 26279566

[B63] Shen-Orr S. S. Milo R. Mangan S. Alon U. (2002). Network motifs in the transcriptional regulation network of *Escherichia coli*. *Nat. Genet.* 31 64–68. 10.1038/ng881 11967538

[B64] Siryaporn A. Goulian M. (2008). Cross-talk suppression between the CpxA-CpxR and EnvZ-OmpR two-component systems in E-coli. *Mol. Microbiol.* 70 494–506. 10.1111/j.1365-2958.2008.06426.x 18761686 PMC2761842

[B65] Skerker J. M. Perchuk B. S. Siryaporn A. Lubin E. A. Ashenberg O. Goulian M. (2008). Rewiring the specificity of two-component signal transduction systems. *Cell* 133 1043–1054. 10.1016/j.cell.2008.04.040 18555780 PMC2453690

[B66] Sneppen K. Krishna S. Semsey S. (2010). Simplified models of biological networks. *Annu. Rev. Biophys.* 39 43–59. 10.1146/annurev.biophys.093008.131241 20192769

[B67] Soo V. W. Wood T. K. (2013). Antitoxin MqsA represses curli formation through the master biofilm regulator CsgD. *Sci. Rep.* 3:3186. 10.1038/srep03186 24212724 PMC4894380

[B68] Stern S. Powers T. Changchien L. M. Noller H. F. (1989). RNA-protein interactions in 30S ribosomal subunits: folding and function of 16S rRNA. *Science* 244 783–790. 10.1126/science.2658053 2658053

[B69] Suel G. M. Garcia-Ojalvo J. Liberman L. M. Elowitz M. B. (2006). An excitable gene regulatory circuit induces transient cellular differentiation. *Nature* 440 545–550. 10.1038/nature04588 16554821

[B70] Swain P. S. Siggia E. D. (2002). The role of proofreading in signal transduction specificity. *Biophys. J.* 82 2928–2933. 10.1016/S0006-3495(02)75633-6 12023215 PMC1302080

[B71] Tabor H. Tabor C. W. (1964). Spermidine, spermine, and related amines. *Pharmacol. Rev.* 16 245–300. 14211123

[B72] Tagkopoulos I. Liu Y. C. Tavazoie S. (2008). Predictive behavior within microbial genetic networks. *Science* 320 1313–1317. 10.1126/science.1154456 18467556 PMC2931280

[B73] Thompson J. F. Hearst J. E. (1983). Structure-function relations in E. coli 16S RNA. *Cell* 33 19–24. 6380748 10.1016/0092-8674(83)90330-6

[B74] Thorner J. W. Paulus H. (1973). Catalytic and allosteric properties of glycerol kinase from *Escherichia coli*. *J. Biol. Chem.* 248 3922–3932. 10.1016/s0021-9258(19)43821-0 4575199

[B75] Venturelli O. S. Zuleta I. Murray R. M. El-Samad H. (2015). Population diversification in a yeast metabolic program promotes anticipation of environmental shifts. *PLoS Biol.* 13:e1002042. 10.1371/journal.pbio.1002042 25626086 PMC4307983

[B76] Wang J. Atolia E. Hua B. Savir Y. Escalante-Chong R. Springer M. (2015). Natural variation in preparation for nutrient depletion reveals a cost-benefit tradeoff. *PLoS Biol.* 13:e1002041. 10.1371/journal.pbio.1002041 25626068 PMC4308108

[B77] Wittkopp P. J. Kalay G. (2011). Cis-regulatory elements: molecular mechanisms and evolutionary processes underlying divergence. *Nat. Rev. Genet.* 13 59–69. 10.1038/nrg3095 22143240

[B78] Wray G. A. (2007). The evolutionary significance of cis-regulatory mutations. *Nat. Rev. Genet.* 8 206–216. 10.1038/nrg2063 17304246

[B79] Wunderlich Z. Mirny L. A. (2009). Different gene regulation strategies revealed by analysis of binding motifs. *Trends Genet.* 25 434–440. 10.1016/j.tig.2009.08.003 19815308 PMC3697852

[B80] Zaslaver A. Mayo A. Ronen M. Alon U. (2006b). Optimal gene partition into operons correlates with gene functional order. *Phys. Biol.* 3 183–189. 10.1088/1478-3975/3/3/003 17021382

[B81] Zaslaver A. Bren A. Ronen M. Itzkovitz S. Kikoin I. Shavit S. (2006a). A comprehensive library of fluorescent transcriptional reporters for *Escherichia coli*. *Nat. Methods* 3 623–628. 10.1038/nmeth895 16862137

[B82] Zinser E. R. Kolter R. (2004). *Escherichia coli* evolution during stationary phase. *Res. Microbiol.* 155 328–336. 10.1016/j.resmic.2004.01.014 15207864

[B83] Zwaig N. Kistler W. S. Lin E. C. (1970). Glycerol kinase, the pacemaker for the dissimilation of glycerol in *Escherichia coli*. *J. Bacteriol.* 102 753–759. 10.1128/jb.102.3.753-759.1970 4914079 PMC247623

[B84] Zwaig N. Lin E. C. (1966). Feedback inhibition of glycerol kinase, a catabolic enzyme in *Escherichia coli*. *Science* 153 755–757. 10.1126/science.153.3737.755 5328677

