# Supplementary material for: Experimental Evolution of Anticipatory Regulation in Escherichia coli
Source: Front Microbiol. 2022 Jan 11;12:796228. doi: 10.3389/fmicb.2021.796228 (PMC8787300; doi:10.3389/fmicb.2021.796228)
Supplement: Supplementary file 1 [file Data_Sheet_1.pdf]

Experimental evolution of anticipatory regulation in *E. coli*.

S1. Details of the rhamnose utilization system, and oxidative stress response strategy in *E. coli*

E. coli utilizes the transporter RhaT to internalize rhamnose (Baldoma et al., 1990a;Garciamartin et al., 1992). Thereafter, the sugar is processed by metabolic enzymes RhaB, RhaA, and RhaD (Moralejo et al., 1993). The regulon is controlled by two AraC-like transcription factors, RhaR and RhaS (Holcroft and Egan, 2000;Wickstrum and Egan, 2004;Wickstrum et al., 2007). Both bind upstream of the coding sequence of the genes essential for rhamnose utilization. The fact that rhamnose utilization regulon has two regulators and that the coding regions of one of the transcription factors (RhaR) and the transporter (RhaT) overlap make the rhamnose utilization regulon unique in *E. coli* (Baldoma et al., 1990b;Via et al., 1996;Wickstrum and Egan, 2004). The rhamnose-utilization regulon is also under catabolite repression, when glucose is present in the environment (Egan and Schleif, 1993).

Prokaryotes protect themselves against oxidative stress (H_2O_2, $^{\cdot}OH$ free radical, $^{\cdot}O^{2-}$) with the action of superoxide dismutases, catalases, and peroxiredoxin (Lushchak, 2001). In the presence of oxidative stress, the expression of these enzymes is up-regulated by transcription factors, OxyR (Zheng et al., 1998), SoxS, and SoxR (Nunoshiba et al., 1992). In a recent study, more than 50 distinct binding sites were reported for OxyR, SoxS and SoxR, when *E. coli* was studied under paraquat stress (Seo et al., 2015). Most of these binding sites were found to be RpoD (Sigma 70)-dependent, and combined, the three transcription factors controlled more than 100 genes in response to oxidative stress. The OxyR, SoxRS regulon includes activation of *zwf* (which encodes glucose-6 phosphate-1-dehydrogenase) , increase in cellular NADPH pool (Henard et al., 2010;Seo et al., 2015), aromatic amino acid production, and upregulation in cell wall biosynthesis (Pomposiello and Demple, 2002).

S2. Range of values of parameters used in the simulation.

Parameter	Value/Range
b (for all promoters)	0 to 0.3
k_on (for all DNA-protein interactions)	0 to 60
k_off (for all DNA-protein interactions)	0.3
kd (degradation rate constant)	0 to 0.015
Signal (S1 or S2)	0 or 1
b_max (maximum benefit)	1.5
Km (half-maximum benefit)	15
co (cost of synthesis of one protein molecule)	0.01

S3. Prior exposure of Alt lines to rhamnose leads to a faster soxS induction.

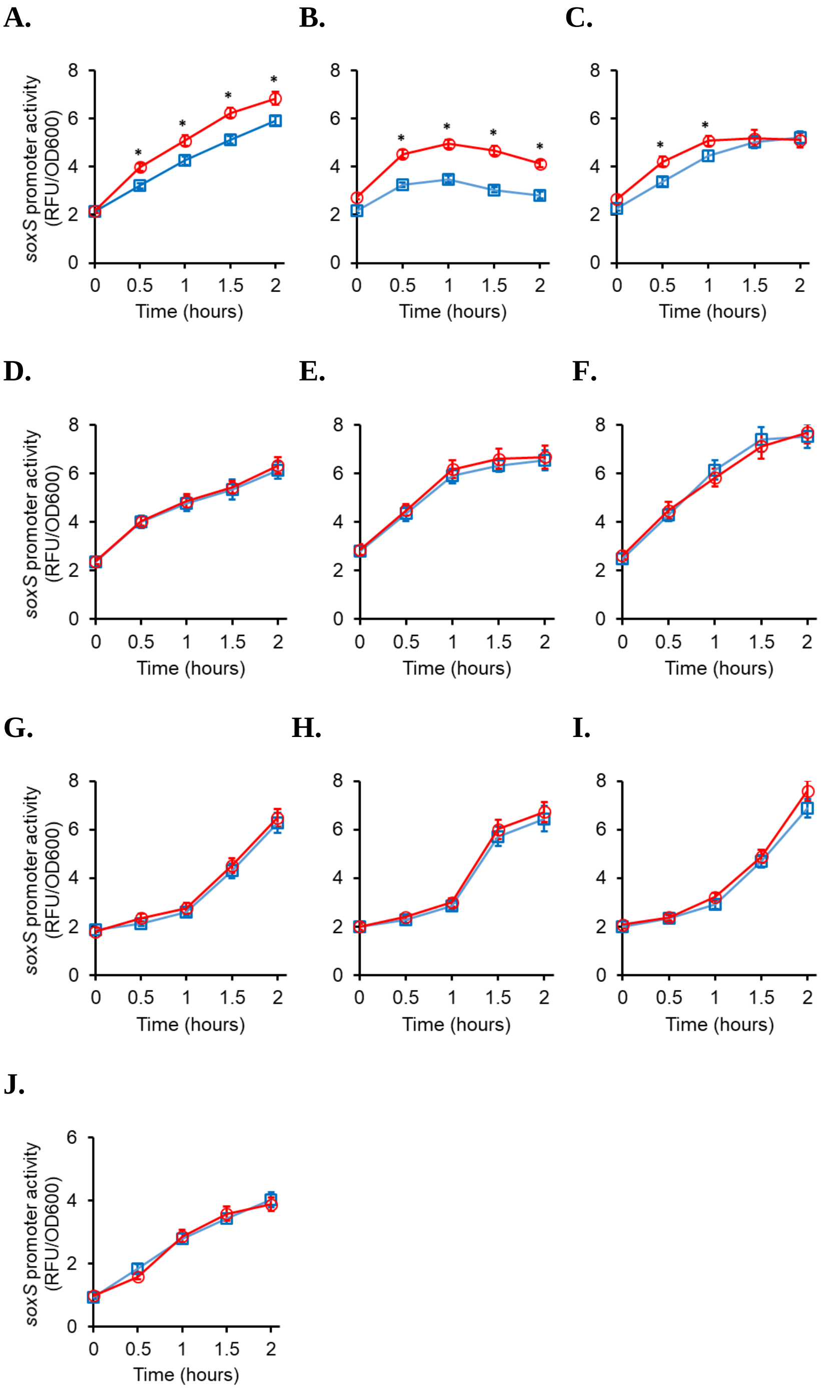

Figure. In the presence of paraquat, lines Alt1 **(A)**, Alt2 **(B)**, and Alt3 **(C)** exhibited a faster induction of the *soxS* promoter when brought from M9 glycerol media containing rhamnose (red) compared to cells brought from M9 glycerol media (blue). The rhamnose-dependent faster induction of the *soxS* promoter is absent in the paraquat-evolved lines (PQ1 **(D)**, PQ2 **(E)**, and PQ3 **(F)**) and in rhamnose-evolved lines (Rha1 **(G)**, Rha2 **(H)**, and Rha3 **(I)**). All experiments were done in triplicate. The average of the three experiments and the standard deviation is reported. **(J)** Ancestor cells, when transitioned to M9 glycerol with paraquat from M9 glycerol media with (red) or without rhamnose (blue) do not exhibit any statistically significant difference in the induction kinetics from the *soxS* promoter. (***** indicates p-value < 0.01, Two-tailed, paired, t-test)

S4. List of mutations in the three Rhamnose-PQ alternating lines.

**Line Alt1**		
	Gene name	Mutation
1.	*potA*	Frameshift mutation (c.764dupA p.Asn255fs)
2.	*trkH*	Missense mutation. (251G>A. Gly84Asp)
3.	*glpK*	Missense mutation. (175T>C. Ser59Pro)
**Line Alt2**		
	Gene name	Mutation
1.	*rpoS*	Missense mutation (377G>A. Gly126Glu)
2.	*ygeH*	Missense mutation. (713A>C. Glu238Ala)
3.	*glpK*	Missense mutation. (184G>T. Val62Leu)
**Line Alt3**		
	Gene name	Mutation
1.	*cyaA*	Frameshift mutation (c.1436_1442delATCAGCC p.His479fs))
2.	*rrsA*	Noncoding transcript variant
3.	*glpK*	Missense mutation. (973A>C. Asn325His)

S5. Codes for simulations.

```
########################################### MAIN FILE ###########################################

for i=1:1:1
maxb = 6;
kmtp = 15;
kona1 = random('Uniform',60,60);
koffa1 = random('Uniform',0.3,0.3);
kona = random('Uniform',60,60);
koffa = random('Uniform',0.3,0.3);
a(i,1) = 0 + (i-1)*0.001;
% a(i,1) = 0.016;
c0 = 0.01;
[fit,bs,ks] = Random(maxb,kmtp,c0,a(i),kona1,koffa1,kona,koffa);
fitness(i,1) = fit(1);
fitness(i,2) = fit(2);
b(i,1) = bs(1);
b(i,2) = bs(2);
b(i,3) = bs(3);
k(i,1) = ks(1);
k(i,2) = ks(2);
k(i,3) = ks(3);
k(i,4) = ks(4);

con(i,1) = 0;
if(fitness(i,1)<fitness(i,2))
    con(i) = 1;
end

end

% plot(a,con,'o');

% [X,Y] = meshgrid(0:0.005:0.3,0:0.005:0.3);
% for i = 1:1:61
%     for j=1:1:61
%         x = X(i,j);
%         y = Y(i,j);
%         Z(i,j) = Simple_Signal_Target2(maxb,kmtp,c0,a,x,y,60,0.3,60,0.3,0.01,1,1,kona1,koffa1,kona,koffa)
- fit(2);
%     end
% end
% surf(X,Y,Z);
% xlabel('b1');
% ylabel('b2');
% zlabel('δF');
% %title('');
% parameters = 'test.mat';
% save(parameters)
% direction = [1 0 0];
% rotate(hsurface,direction,25)




########################################### Function file. Name → Random
###########################################

function [r,bs,kc] = Random(maxb,kmtp,c0,a,kona1,koffa1,kona,koffa)
maxf = zeros(2,1);
rtotal = 0.01;
s1 = 1;
s = 1;
bs = zeros(3,1);
kc = zeros(4,1);

for kondna = 60:1:60
    for koffdna = 0.3:0.1:0.3
        for bn = 0:0.005:0.3
            fitness = Simple_Signal_Target(maxb,kmtp,c0,a,bn,kondna,koffdna,rtotal,s,kona,koffa);
            if(fitness>maxf(1))
                maxf(1) = fitness;
                bs(1) = bn;
            end
        end
    end
end

c = 1;
for kondna1 = 60:1:60
    for koffdna1 = 0.3:0.1:0.3
        for kondna = 60:1:60
            for koffdna = 0.3:0.005:0.3
```

```
                for b1 = 0:0.005:0.3
                    for bc = 0:0.005:0.3
                        fitness =
Simple_Signal_Target2(maxb,kmtp,c0,a,b1,bc,kondna1,koffdna1,kondna,koffdna,rtotal,s1,s,kona1,koffa1,kona,ko
ffa);
                        % k1k2fit(c,:) = [b1 bc fitness];
                        % c = c+1;
                        if(fitness>maxf(2))
                            maxf(2) = fitness;
                            bs(2) = b1;
                            bs(3) = bc;
                            kc = [kondna1;koffdna1;kondna;koffdna];
                        end
                    end
                end
            end
        end
    end
end


%plot(a,bs(2)','*');
%hold on;
%plot(a,bs(3),'.');
%xlabel('degradation rate α');
%ylabel('Transcription rate b');
r = maxf;
%legend('b1','b2');
```

```
############################################ Function file → No conditioning case
############################################

function answer2 = Simple_Signal_Target(maxb,kmtp,c0,a,b,kondna,koffdna,rtotal,s,kona,koffa)
%Target Protein 2
n = 1;                %no. of signal molecules binding to activator protein
%s = 0.005;             %signal concentration
%kona = 100;           %k_on of signal-activator complex
%koffa = 0.2;            %k_off of signal-activator complex
kxa = koffa/kona;     %ratio
%rtotal = 10;          %total concentration of activator protein

%main parameters
%b = 1.5;                %maximal translation rate
%a = 0.08;               %degradation rate of target protein
%maxb = 4;               %maximal benefit conferred by target protein
%kmtp = 10;              %concentration at which half-maximal benefit is conferred
%c0 = 0.5;               %cost of production for one cell
a1 = 1;                 %no. of proteins produced by cell per unit time

t = zeros(200,1);       %time
tspan = zeros(200,1);
for i=1:1:200
    t(i) = i;
    tspan(i) = i;
end
ract_steady = ((s^n)*rtotal)/(kxa + (s^n)); %concentration of activated protein at steady state
ract = ract_steady*(1 - exp(-koffa*t*(kxa+s))); %concentration of activated protein

%koffdna = 0.1;       %k_off of dna-activator complex
%kondna = 100;        %k_on of dna-activator complex
kxdna = koffdna/kondna;

[x,prob] = ode23s(@(x,prob) kondna*ract_steady*(1-exp(-koffa*(kxa+s)*x))*(1-prob) - koffdna*prob, tspan,
0);
pv = prob(200);
%solve the ode, to get tp, as a function of t
p = polyfit(x,prob,3);

%p(1)*x^3 + p(2)*x^2 + p(3)*x + p(4)
[x,tp] = ode23s(@(x,tp) b*(p(1)*x^3 + p(2)*x^2 + p(3)*x + p(4)) - a*tp, tspan,0);
% plot(t,ben,'r');
% hold on;
% plot(t,cost,'b');
%overall cost
tp_total = 0;


for i=1:1:4                     %dividing into five segments
    tf = (i*t(200)/4);          %upper limit of adaptive quadrature
    ti = 1 + ((i-1)*t(200)/4);  %lower limit
    tc(1) = ti;                 %points at which values are calculated
    tc(5) = tf;
    tc(3) = (ti+tf)/2;
```

```
    tc(2) = (ti+tc(3))/2;
    tc(4) = (tc(3)+tf)/2;
    rop1 = zeros(5,1);           %amount of protein at different times
    for j=1:1:5
        tc(j) = round(tc(j));
        rop1(j) = prob(tc(j))*b;  %calculating rate of production at each point
    end
    tp1 = ((tf-ti)/6)*(rop1(1)+4*rop1(3)+rop1(5));
    tp2 = ((tf-ti)/12)*(rop1(1)+4*rop1(2)+2*rop1(3)+4*rop1(4)+rop1(5));
    ptp = 16*tp2/15 - tp1/15; %total in this segment
    tp_total = tp_total + ptp;      %summing up all five segments
end
ocost = tp_total*c0/a1;

%cost after signal stops
extrat = 1;
[x,prob] = ode23s(@(x,prob) kondna*ract(200)*exp(-koffa*x)*(1-prob) - koffdna*prob, tspan, pv);
tspane = 0;
for j=1:1:200
    rop_f = prob(j)*b;
    t(200+j) = 200+j;
    if(rop_f<0.001)
        extrat = j;
        for k=1:1:j
            tspane(k) = k;
        end
        break
    end
end
if(size(tspane)<2)
    for i=1:1:200
        tspane(i) = i;
    end
end

[x,prob] = ode23s(@(x,prob) kondna*ract(200)*exp(-koffa*x)*(1-prob) - koffdna*prob, tspane, pv);
p = polyfit(x,prob,3);
y = polyval(p,x);

[x,tpe] = ode23s(@(x,tpe) b*(p(1)*x^3 + p(2)*x^2 + p(3)*x + p(4)) - a*tpe, tspan,tp(200));
for j=1:1:extrat
    tp(200+j) = tpe(j);
end

tf = extrat;
ti = 1;
tc(1) = ti;
tc(5) = tf;
tc(3) = (ti+tf)/2;
tc(2) = (ti+tc(3))/2;
tc(4) = (tc(3)+tc(5))/2;
rope = zeros(5,1);     %target protein concentration after signal stops
for i=1:1:5
    tc(i) = round(tc(i));
    rope(i) = prob(tc(i))*b; %rate of production after signal stops at all 5 values
end
tpe1 = ((tf-ti)/6)*(rope(1)+4*rope(3)+rope(5));
tpe2 = ((tf-ti)/12)*(rope(1)+4*rope(2)+2*rope(3)+4*rope(4)+rope(5));
tp_ex = 16*tpe2/15 - tpe1/15; % total target protein produced after signal stopping
coste = tp_ex*c0/a1;     %extra cost after signal stops
ocost = ocost + coste;

%overall benefit
oben = 0;
for i=1:1:4            %dividing into 5 segments
    tf = i*t(200)/4;    %final point
    ti = 1 + ((i-1)*t(200)/4);%initial point
    tc(1) = ti;
    tc(5) = tf;
    tc(3) = (ti+tf)/2;
    tc(2) = (ti+tc(3))/2;
    tc(4) = (tc(3)+tf)/2;
    pben = zeros(5,1); %pseudo benefit, to store benefit of each segment
    for j=1:1:5
        tc(j) = round(tc(j));
        pben(j) = maxb*tp(tc(j))/(kmtp + tp(tc(j)));
    end
    pben1 = ((tf-ti)/6)*(pben(1)+4*pben(3)+pben(5));
    pben2 = ((tf-ti)/12)*(pben(1)+4*pben(2)+2*pben(3)+4*pben(4)+pben(5));
    sben = 16*pben2/15 - pben1/15;%benefit conferred in this segment
    oben = oben + sben;           %summing up all benefits conferred at all times
end
oben = oben/200;
answer2 = oben - ocost;
```

```
############################################ Function file → Conditioning present case
############################################

function answer =
Simple_Signal_Target2(maxb,kmtp,c0,a,b1,b,kondna1,koffdna1,kondna,koffdna,rtotal,s1,s,kona1,koffa1,kona,kof
fa)
n = 1;                %no. of signal molecules binding to activator protein
%rtotal = 10;         %total concentration of activator protein

%s1 = 0.005;          %signal concentration
% a = 0.08;           %degradation rate of target protein
% maxb = 4;           %maximal benefit conferred by target protein
% kmtp = 15;          %concentration at which half-maximal benefit is conferred
% c0 = 0.5;           %cost of production for one cell
a1 = 1;               %no. of proteins produced by cell per unit time

%kona1 = 60;          %k_on of signal-activator complex
%koffa1 = 0.3;        %k_off of signal-activator complex
kxa = koffa1/kona1;   %ratio


%main parameters
% b = 0.7;            %maximal translation rate
% koffdna1 = 0.3;     %k_off of dna-activator complex
% kondna1 = 10;       %k_on of dna-activator complex
kxdna1 = koffdna1/kondna1;
tspan = zeros(200,1);
t = zeros(200,1);     %time
for i=1:1:200
    t(i) = i;
    tspan(i) = i;
end
ract_steady = ((s1^n)*rtotal)/(kxa + (s1^n));    %concentration of activated protein at steady state
ract = ract_steady*(1 - exp(-koffa1*(kxa+s)*t)); %concentration of activated protein
ract1 = ract(200);
[x,prob] = ode23s(@(x,prob) kondna1*ract_steady*(1-exp(-koffa1*(kxa+s)*x))*(1-prob) - koffdna1*prob, tspan,
0);
%solve the ode, to get tp, as a function of t
p = polyfit(x,prob,3);
y = polyval(p,x);
pv = prob(200);

%solve the ode, to get tp, as a function of t
[x,tp] = ode23s(@(x,tp) b1*(p(1)*x^3 + p(2)*x^2 + p(3)*x + p(4)) - a*tp, tspan,0);
% plot(t,ben,'r');
% hold on;
% plot(t,cost,'b');
%overall cost
tp_total = 0;

for i=1:1:4                     %dividing into five segments
    tf = (i*t(200)/4);          %upper limit of adaptive quadrature
    ti = 1 + ((i-1)*t(200)/4);  %lower limit
    tc(1) = ti;                 %points at which values are calculated
    tc(5) = tf;
    tc(3) = (ti+tf)/2;
    tc(2) = (ti+tc(3 ))/2;
    tc(4) = (tc(3)+tf)/2;
    rop1 = zeros(5,1);           %amount of protein at different times
    for j=1:1:5
        tc(j) = round(tc(j));
        rop1(j) = prob(tc(j))*b1;%calculating rate of production at each point
    end
    tp1 = ((tf-ti)/6)*(rop1(1)+4*rop1(3)+rop1(5));
    tp2 = ((tf-ti)/12)*(rop1(1)+4*rop1(2)+2*rop1(3)+4*rop1(4)+rop1(5));
    ptp = 16*tp2/15 - tp1/15;    %total in this segment
    tp_total = tp_total + ptp;   %summing up all five segments
end
ocost = tp_total*c0/a1;

answer1 = -ocost;



%SIGNAL2
n = 1;                   %no. of signal molecules binding to activator protein
%s = 0.005;              %signal concentration
%kona = 100;             %k_on of signal-activator complex
%koffa = 0.2;            %k_off of signal-activator complex
kxa = koffa/kona;        %ratio
%rtotal = 10;            %total concentration of activator protein

%main parameters
%b = 1.5;                %maximal translation rate

t = zeros(200,1);        %time
```

```
tspan = zeros(200,1);

for i=1:1:200
    t(i) = i;
    tspan(i) = i;
end
ract_f = ract(200);
ract_steady = ((s^n)*rtotal)/(kxa + (s^n)); %concentration of activated protein at steady state
ract = ract_steady*(1 - exp(-koffa*(kxa+s)*t)); %concentration of activated protein

% koffdna = 0.1;       %k_off of dna-activator complex
% kondna = 100;        %k_on of dna-activator complex
kxdna = koffdna/kondna;

[x,prob] = ode23s(@(x,prob) kondna*ract_steady*(1-exp(-koffa*(kxa+s)*x))*(1-prob) - koffdna*prob, tspan,
0);
p = polyfit(x,prob,3);
y = polyval(p,x);
pv2 = prob(200);
prob2 = prob;
[x,prob] = ode23s(@(x,prob) kondna1*ract1*exp(-koffa1*x)*(1-prob) - koffdna1*prob, tspan, pv);
p1 = polyfit(x,prob,3);
y1 = polyval(p1,x);
prob1 = prob;

% %solve the ode, to get tp, as a function of t

[x,tps2] = ode23s(@(x,tps2) b*(p(1)*x^3 + p(2)*x^2 + p(3)*x + p(4)) + b1*(p1(1)*x^3 + p1(2)*x^2 + p1(3)*x +
p1(4))  - a*tps2, tspan,tp(200));

for i=1:1:200
    tp(i+200) = tps2(i);
end

tp_total = 0;
for i=1:1:4                     %dividing into five segments
    tf = (i*t(200)/4);          %upper limit of adaptive quadrature
    ti = 1 + ((i-1)*t(200)/4);  %lower limit
    tc(1) = ti;                 %points at which values are calculated
    tc(5) = tf;
    tc(3) = (ti+tf)/2;
    tc(2) = (ti+tc(3))/2;
    tc(4) = (tc(3)+tf)/2;
    rop1 = zeros(5,1);
    rop2 = zeros(5,1);%amount of protein at different times
    for j=1:1:5
        tc(j) = round(tc(j));
        rop1(j) = prob1(tc(j))*b1;  %calculating rate of production at each point
        rop2(j) = prob2(tc(j))*b;
        rop1(j) = rop1(j) + rop2(j);
    end
    tp1 = ((tf-ti)/6)*(rop1(1)+4*rop1(3)+rop1(5));
    tp2 = ((tf-ti)/12)*(rop1(1)+4*rop1(2)+2*rop1(3)+4*rop1(4)+rop1(5));
    ptp = 16*tp2/15 - tp1/15;       %total in this segment
    tp_total = tp_total + ptp;      %summing up all five segments
end
ocost = tp_total*c0/a1;

%cost after signal stops
extrat = 1;
[x,prob] = ode23s(@(x,prob) kondna*ract(200)*exp(-koffa*x)*(1-prob) - koffdna*prob, tspan, pv2);
tspane = 0;
for j=1:1:200
    rop_f = prob(j)*b;
    t(200+j) = 200+j;
    if(rop_f<0.001) %only till concentrations where cost is significant; not required after production ~0
        extrat = j;
        for k=1:1:j
            tspane(k) = k;
        end
        break
    end
end
if(size(tspane)<2)
    for i=1:1:200
        tspane(i) = i;
    end
end

[x,prob] = ode23s(@(x,prob) kondna*ract(200)*exp(-koffa*x)*(1-prob) - koffdna*prob, tspane, pv2);
p = polyfit(x,prob,3);
y = polyval(p,x);


[x,tpe] = ode23s(@(x,tpe) b*(p(1)*x^3 + p(2)*x^2 + p(3)*x + p(4)) - a*tpe, tspan,tp(400));
for j=1:1:extrat
    tp(400+j) = tpe(j);
end
```

```
tf = extrat;
ti = 1;
tc(1) = ti;
tc(5) = tf;
tc(3) = (ti+tf)/2;
tc(2) = (ti+tc(3))/2;
tc(4) = (tc(3)+tc(5))/2;
rope = zeros(5,1);           %target protein concentration after signal stops
for i=1:1:5
    tc(i) = round(tc(i));
    rope(i) = prob(tc(i))*b; %rate of production after signal stops at all 5 values
end
 tpe1 = ((tf-ti)/6)*(rope(1)+4*rope(3)+rope(5));
 tpe2 = ((tf-ti)/12)*(rope(1)+4*rope(2)+2*rope(3)+4*rope(4)+rope(5));
 tp_ex = 16*tpe2/15 - tpe1/15;% total target protein produced after signal stopping
 coste = tp_ex*c0/a1;         %extra cost after signal stops
 ocost = ocost + coste;

%overall benefit
oben = 0;
for i=1:1:4                   %dividing into 5 segments
    tf = i*t(200)/4;          %final point
    ti = 1 + ((i-1)*t(200)/4);%initial point
    tc(1) = ti;
    tc(5) = tf;
    tc(3) = (ti+tf)/2;
    tc(2) = (ti+tc(3))/2;
    tc(4) = (tc(3)+tf)/2;
    pben = zeros(5,1);        %pseudo benefit, to store benefit of each segment
    for j=1:1:5
        tc(j) = round(tc(j));
        pben(j) = maxb*tps2(tc(j))/(kmtp + tps2(tc(j)));
    end
    pben1 = ((tf-ti)/6)*(pben(1)+4*pben(3)+pben(5));
    pben2 = ((tf-ti)/12)*(pben(1)+4*pben(2)+2*pben(3)+4*pben(4)+pben(5));
    sben = 16*pben2/15 - pben1/15;%benefit conferred in this segment
    oben = oben + sben;           %summing up all benefits conferred at all times
end
oben = oben/200;
answer2 = oben - ocost;
ocost = ocost - answer1;

for i=1:1:(400+extrat)
    t(i)= i;
end
answer = answer1 + answer2;


################################################### END ###################################################
```

S6. Supplement References

Baldoma, L., Badia, J., Sweet, G., and Aguilar, J. (1990a). Cloning, Mapping and Gene-Product Identification of Rhat from Escherichia-Coli K12. *Fems Microbiology Letters* 72**,** 103-108.

Baldoma, L., Badia, J., Sweet, G., and Aguilar, J. (1990b). Cloning, mapping and gene product identification of rhaT from Escherichia coli K12. *FEMS Microbiol Lett* 60**,** 103-107.

Egan, S.M., and Schleif, R.F. (1993). A Regulatory Cascade in the Induction of Rhabad. *Journal of Molecular Biology* 234**,** 87-98.

Garciamartin, C., Baldoma, L., Badia, J., and Aguilar, J. (1992). Nucleotide-Sequence of the Rhar-Soda Interval Specifying Rhat in Escherichia-Coli. *Journal of General Microbiology* 138**,** 1109-1116.

Henard, C.A., Bourret, T.J., Song, M., and Vazquez-Torres, A. (2010). Control of Redox Balance by the Stringent Response Regulatory Protein Promotes Antioxidant Defenses of Salmonella. *Journal of Biological Chemistry* 285**,** 36785-36793.

Holcroft, C.C., and Egan, S.M. (2000). Interdependence of activation at rhaSR by cyclic AMP receptor protein, the RNA polymerase alpha subunit C-terminal domain, and rhaR. *J Bacteriol* 182**,** 6774-6782.

Lushchak, V.I. (2001). Oxidative stress and mechanisms of protection against it in bacteria. *Biochemistry-Moscow* 66**,** 476-489.

Moralejo, P., Egan, S.M., Hidalgo, E., and Aguilar, J. (1993). Sequencing and Characterization of a Gene-Cluster Encoding the Enzymes for L-Rhamnose Metabolism in Escherichia-Coli. *Journal of Bacteriology* 175**,** 5585-5594.

Nunoshiba, T., Hidalgo, E., Amabile Cuevas, C.F., and Demple, B. (1992). Two-stage control of an oxidative stress regulon: the Escherichia coli SoxR protein triggers redox-inducible expression of the soxS regulatory gene. *J Bacteriol* 174**,** 6054-6060.

Pomposiello, P.J., and Demple, B. (2002). Global adjustment of microbial physiology during free radical stress. *Advances in Microbial Physiology, Vol 46* 46**,** 319-341.

Seo, S.W., Kim, D., Szubin, R., and Palsson, B.O. (2015). Genome-wide Reconstruction of OxyR and SoxRS Transcriptional Regulatory Networks under Oxidative Stress in Escherichia coli K-12 MG1655. *Cell Reports* 12**,** 1289-1299.

Via, P., Badia, J., Baldoma, L., Obradors, N., and Aguilar, J. (1996). Transcriptional regulation of the Escherichia coli rhaT gene. *Microbiology* 142 (Pt 7)**,** 1833-1840.

Wickstrum, J.R., and Egan, S.M. (2004). Amino acid contacts between sigma 70 domain 4 and the transcription activators RhaS and RhaR. *J Bacteriol* 186**,** 6277-6285.

Wickstrum, J.R., Skredenske, J.M., Kolin, A., Jin, D.J., Fang, J., and Egan, S.M. (2007). Transcription activation by the DNA-binding domain of the AraC family protein RhaS in the absence of its effector-binding domain. *J Bacteriol* 189**,** 4984-4993.

Zheng, M., Aslund, F., and Storz, G. (1998). Activation of the OxyR transcription factor by reversible disulfide bond formation. *Science* 279**,** 1718-1721.
